# Aerobic Exercise Delays Alzheimer’s Disease by Regulating Mitochondrial Proteostasis in the Cerebral Cortex and Hippocampus

**DOI:** 10.3390/life13051204

**Published:** 2023-05-17

**Authors:** Kaiyin Cui, Chaoyang Li, Guoliang Fang

**Affiliations:** China Institute of Sport Science, Beijing 100061, China

**Keywords:** aerobic exercise, mitochondrial proteostasis, mitochondrial unfolded protein reaction, mitochondrial autophagy, mitochondrial protein import, Alzheimer’s disease

## Abstract

In clinical practice, Alzheimer’s disease (AD), as one of the main neurodegenerative diseases globally, currently has no cure. Recently, the delaying and improving effects of physical exercise on AD have gradually been confirmed; however, the specific mechanism involved needs further clarification. (1) Objective: Explore the mechanism aerobic exercise plays in delaying AD by regulating mitochondrial proteostasis and provide new theoretical bases for improving and delaying AD through aerobic exercise in the future. (2) Methods: Male APP/PS1 mice were randomly divided into a normal group (NG, n = 20), activation group (AG, n = 20), and inhibition group (SG, n = 20). Then, the mice in each group were randomly divided into control group and exercise group (n = 10 mice each), yielding the normal control group (CNG), normal exercise group (ENG), active control group (CAG), active exercise group (EAG), inhibitive control group (CSG), and inhibitive exercise group (ESG). After adaptive training, the mice in the exercise groups were trained on an aerobic treadmill for 12 weeks; we conducted behavioral tests and sampled the results. Then, quantitative real-time PCR (Q-PCR) and Western blot analysis were performed. (3) Results: In the Morris water maze (MWM) test, the latency was significantly reduced and the number of platform crossings was significantly increased in the CAG and ENG compared with the CNG, while the result of the CSG was contrary to this. Compared with the ENG, latency was significantly reduced and the number of platform crossings was significantly increased in the EAG, while the opposite occurred for ESG. Compared with the CAG, the latency was significantly reduced and the number of platform crossings was significantly increased in the EAG, while the results for CSG were contrary. In the step-down test, compared with the CNG, the latency was significantly increased and the number of errors was significantly reduced in the CAG and ENG, respectively, while the results for CSG were contrary. Compared with the ENG, the latency was significantly increased and the number of errors was significantly reduced in the EAG, while the results for ESG were contrary. Compared with the CAG, the latency was significantly increased and the number of errors was significantly reduced in the EAG, while the results for CSG were contrary. Mitochondrial unfolded protein reactions (UPR^mt^), mitochondrial autophagy, and mitochondrial protein import levels in each group of mice were detected using Q-PCR and Western blot experiments. Compared with the CNG, the UPR^mt^ and mitochondrial autophagy levels in the CAG and ENG were significantly increased and the mitochondrial protein import levels were significantly reduced, while the results for the CSG were contrary. Compared with the ENG, the UPR^mt^ and mitochondrial autophagy levels in the EAG were significantly increased and the mitochondrial protein import levels were significantly reduced, while the results for ESG were contrary. Compared with the CAG, the UPR^mt^ and mitochondrial autophagy levels in the EAG were significantly increased and the mitochondrial protein import levels were significantly reduced, while the results for CSG were contrary. (4) Conclusions: Aerobic exercise can improve cognitive function levels and delay the symptoms of AD in APP/PS1 mice by regulating mitochondrial proteostasis.

## 1. Introduction

Alzheimer’s disease (AD), a form of dementia, mostly occurs in elderly individuals. Similar to Parkinson’s disease (PD) and Huntington’s disease (HD), it is a degenerative disease of the nervous system and has long represented a great challenge in the field of public health [1]. As a common degenerative disease of the nervous system, AD often occurs with aging. The specific clinical manifestations of the disease are progressive cognitive dysfunction and memory decline. Its pathological characteristics are the appearance of senile plaques (SPS) in the brain; the formation of neurofibrillary tangles (NFTs); a reduction in the number of cerebral cortical cells, such as neurons and microglia; synaptic plasticity changes; and the amyloidosis of local arteries and vessels [2,3]. Mitochondria are highly dynamic organelles that provide energy for cell life activities [4]. Under normal circumstances, mitochondria, with their highly dynamic characteristics, make adaptive changes to intracellular and extracellular stimuli, continuously maintaining their own structural and functional stability, namely, mitochondrial homeostasis [5]. Life activities, such as energy metabolism in the body and cells, are highly dependent on mitochondrial homeostasis [5]. With the continuous extension of research, scholars have proposed the concept of mitochondrial proteostasis as one of the most important components of overall mitochondrial homeostasis. When mitochondria are stimulated by internal and external stimuli, they remove damaged and impaired mitochondria through pathways such as mitochondrial unfolded protein response (UPR^mt^), mitochondrial autophagy, and the regulation of mitochondrial protein transport and mitochondrial protein deacetylation levels, as well as regulating mitochondrial energy metabolism, controlling mitochondrial quality and quantity, and maintaining the mitochondrial dynamic balance [4,5,6]. In recent years, an increasing number of studies have shown that mitochondrial dysfunction is closely related to the occurrence and development of AD. Mitochondria are gradually being recognized and used as targets in the treatment of AD [6,7]. Recently, studies have found that mitochondrial proteostasis is disrupted in the brains of AD patients, leading to mitochondrial dysfunction, which manifests as the dysfunction of UPR^mt^, mitochondrial autophagy, and mitochondrial protein import [6,8,9,10]. Moreover, it has been gradually confirmed that an appropriate amount of exercise can effectively delay and ameliorate neurodegenerative diseases, especially AD [11]. Therefore, this experiment explores the role and mechanism of aerobic exercise in delaying AD by regulating mitochondrial proteostasis in terms of three aspects, namely, UPR^mt^, mitochondrial autophagy, and mitochondrial protein import levels, thus providing new ideas and theoretical bases for the prevention and treatment of AD through exercise in the future.

## 2. Materials and Methods

### 2.1. Animals and Groups

Forty 8-month-old male APP/PS1 double-transgenic mice, weighing 31.24 ± 4.16 g, were housed with 2 mice per cage in a 12 h:12 h light cycle at a temperature of 20 °C ± 2 °C and relative humidity of 40–60% and provided free access to food and water. After one week of adaptive feeding, the APP/PS1 mice were randomly divided into three groups: the normal group (NG, n = 20), the activation group (AG, n = 20), and the inhibition group (SG, n = 20). The AG group received the mitochondrial proteostasis activator nicotinamide ribose (NR) via gavage at a standard of 400 mg/kg body weight per mouse, while the SG group received the mitochondrial proteostasis inhibitor chloroquine via intraperitoneal injection at a standard of 15 mg/kg body weight per mouse, once a day for 13 weeks. Then, the three groups of mice were randomly divided into the control group and the exercise group, with 10 mice in each group. These were subdivided into the normal control group (CNG, n = 10), the normal exercise group (ENG, n = 10), the activation control group (CAG, n = 10), and the activation exercise group (EAG, n = 10), the inhibitive control group (CSG), and the inhibitive exercise group (ESG). The use of NR and mouse models was based on the experimental research of Sorrentino et al. [8]. The use of chloroquine was based on the experimental research of Li et al. [12].

### 2.2. Training Program

The mice in the ENG, EAG, and ESG were trained on a treadmill at a speed of 9 m/min for 10 min a day for 5 days. After resting for 2 days, the two groups of mice underwent formal training for 12 weeks according to the scheme presented in Table 1. The mice were trained 5 days per week (Monday to Friday). The mice in the CNG, CAG, and CSG were kept inactive.

### 2.3. Morris Water Maze (MWM) Test

After 12 weeks of treadmill training, the MWM test was used to test the positioning navigation ability and spatial exploration behavior of the mice in each group and to evaluate the spatial learning capacity and memory of the mice. The test consisted of three phases: the cued learning phase, the place navigation test, and the spatial probe test. The cued learning phase, which consisted of four trials, was performed the day before the place navigation test in order to assess the ability of the mice to swim to a cued target and adapt to the environment. In the cued learning phase, the hidden platform was marked by a “flag” above the water’s surface. The place navigation test was carried out as follows: on each of the first 5 days, the mice were placed in the water in one of the quadrants facing the pool wall and the escape latency, i.e., the time from when the mouse entered the water to when it found the platform, was recorded. If a mouse could not find the platform within 2 min, the experimenter gently guided it to the platform and recorded a latency of 120 s. The intervals between each test were 15 min, and the average of the four latencies was taken as the final score for each day. The spatial probe test was carried out as follows: on the 6th day, the platform was removed, the mice were placed in the water in the same quadrant facing the pool wall, and the time spent swimming in the platform quadrant within 60 s was recorded to evaluate the spatial memory of the mice.

### 2.4. The Step-Down Test

The mice were first placed in a platform diving apparatus and adapted to the environment for 3 min. Then, a 0.2 mA current was delivered to the grid on the bottom of the apparatus, and the latency of the mice jumping onto a rubber pad and the number of electric shocks received within 5 min (the number of errors) were recorded to assess learning. After 24 h, the mice were placed in the diving box and allowed to adapt for 3 min before being placed on the rubber pad. The latency of jumping off the rubber pad for the first time and the number of errors were recorded in order to evaluate memory.

### 2.5. Sample Preparation and Extraction

After the MWM test, all mice were anesthetized intraperitoneally with sodium pentobarbital (50 mg/kg) and then decapitated. The bilateral hippocampus (CA1 area) and ipsilateral cerebral cortex (prefrontal cortex area, PFC area) were separated and placed on ice, and all samples were immediately frozen at −80 °C until analysis could be performed.

### 2.6. Isolation of Mitochondria from Tissue Samples

Cerebral cortex and hippocampus samples from the mice in each group were cut into tissue blocks of the same size. After weighing, they were washed with ice-cold PBS solution, cut into small pieces, and then transferred to an ice-cold glass homogenizer. The tissue blocks were immersed in the mitochondrial separation solution containing protease inhibitor, and homogenized in the ice bath environment for about 10 times. The volume of the mitochondrial isolation solution is approximately ten times the volume of the tissue block. The samples were centrifuged at 600× *g* at 4 °C for 5 min, and the supernatant was collected and centrifuged at 11,000× *g* at 4 °C for 10 min. The supernatant was then discarded, and the isolated mitochondria were precipitated. A Tissue Mitochondria Isolation Kit was purchased from Beyotime.

### 2.7. Quantitative Real-Time PCR (Q-PCR)

An appropriate amount of cerebral cortex and hippocampus tissue from mice in each group was placed into a glass homogenizer. A total of 1 mL of TRIzol lysate was added, and the tissues were fully homogenized and lysed. Total RNA was extracted from the tissue with chloroform/isopropanol and then reverse-transcribed into cDNA with reverse transcriptase. All the above operations were carried out on ice. Primers (Table 2) for Q-PCR were designed based on the sequence of the target gene obtained from the NCBI database. The relative expression of each target gene in each group was calculated from the CT value. (The selection of indicators was based on the experimental research of Sorrentino et al. [8].)

### 2.8. Western Blot Analysis

An appropriate amount of cerebral cortex and hippocampus tissue from mice in each group was placed in a mortar, ground in liquid nitrogen, and then transferred to a 1.5 mL EP tube. Protein lysis buffer was quickly added to lyse the tissue. After full lysis, the tissues were spun in a centrifuge at 12,000× *g* for 10 min, the supernatant was placed in a new EP tube, protein loading buffer was added, and the samples were placed into a 100 °C water bath for protein denaturation for 5 min. Then, SDS-PAGE was conducted. After electrophoresis, the proteins were transferred to a nitrocellulose membrane, blocked with 5% nonfat milk powder (dissolved in TBST) for 1 h at room temperature, and then incubated with diluted primary antibody (diluted in TBST containing 1% nonfat milk powder) overnight at 4 °C. The blots were probed using the following antibodies: HSPA9 (ab2799, 1:1000, Abcam, Cambridge, UK), HSP60 (ab46798, 1:5000, Abcam, Cambridge, UK), HSP10 (ab109624, 1:1000, Abcam, Cambridge, UK), YME1L1 (ab170123, 1:800, Abcam, Cambridge, UK), ClPP (ab124822, 1:1000, Abcam, Cambridge, UK), LONP1 (ab224316, 1:1000, Abcam, Cambridge, UK), BNIP3 (ab109362, 1:1000, Abcam, Cambridge, UK), P62 (ab109012, 1:1000, Abcam, Cambridge, UK), PARKIN (ab77924, 1:2000, Abcam, Cambridge, UK), PINK1 (ab23707, 1:800, Abcam, Cambridge, UK), LC3A (ab52768, 1:2000, Abcam, Cambridge, UK), TIM23 (ab230253, 1:1000, Abcam, Cambridge, UK), TIM17A (ab192246, 1:1000, Abcam, Cambridge, UK), eIF2α (ab169528, Abcam, Cambridge, UK), ATF-1 (ab181569, 1:1000, Abcam, Cambridge, UK), and β-Actin (1:1000, ab8226, Abcam, Cambridge, UK). These antibodies were diluted at the indicated ratio and purchased from the indicated company. The next day, the membrane was washed three times with TBST for 5 min each time to remove the residual primary antibody. Then, the membrane was incubated with diluted secondary antibody (diluted in TBST containing 1% skimmed milk powder) at room temperature for 1 h and then washed four times with TBST for 5 min each time to remove the residual secondary antibody. Finally, ECL reagent and X-ray film were used to visualize the protein signal. ImageJ 1.8.0 software was used to analyze the gray value of the target protein bands. (The selection of indicators was based on the experimental research of Sorrentino et al. [8].)

### 2.9. Data Analysis

Image J software was used to perform the grayscale analysis of the Western blot bands, and SPSS 19.0 software was used to conduct statistical analysis. Normality was assessed using the Shapiro–Wilk test, and homogeneity of variance was assessed using the F-test. If the data met the assumptions of normality and homogeneity of variance, one-way ANOVA with Bonferroni post hoc analysis was used and the means (SD) was used as the descriptive statistics. If the assumptions were not met, the Kruskal–Wallis test was used and the medians (IQR) was used as the descriptive statistics. Origin 8.0 software was used for plotting. *p* < 0.05 was considered statistically significant.

## 3. Results

### 3.1. Aerobic Exercise Improves Cognitive Function in APP/PS1 Mice by Regulating Mitochondrial Proteostasis

#### 3.1.1. MWM

**Figure 1:**
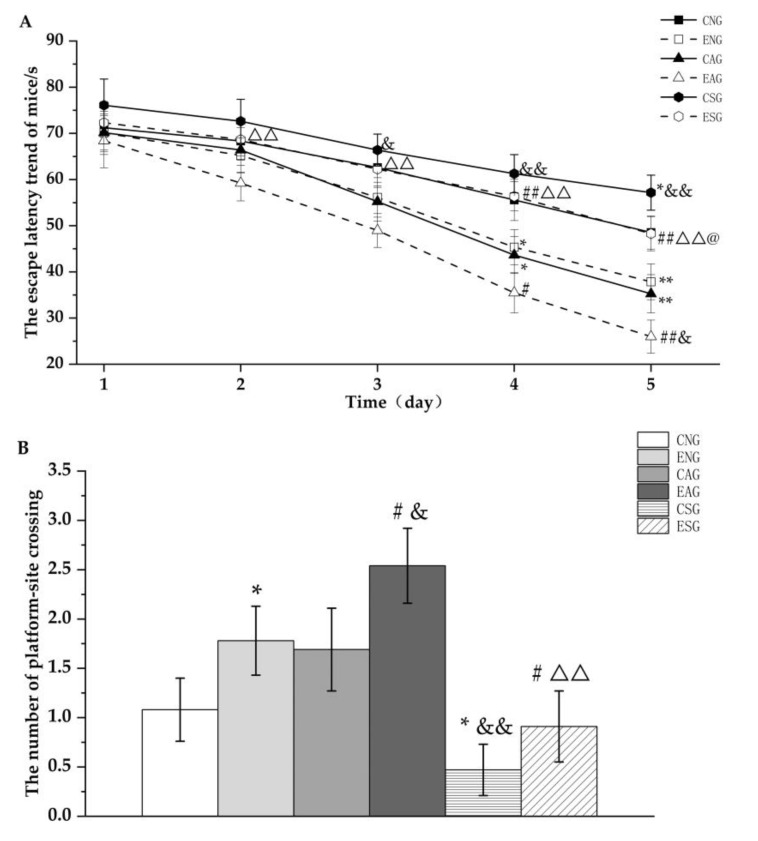
Trend of the escape latency (**A**) and the number of platform crossings (**B**) of the mice in the MWM test. Note: * represents *p* < 0.05 and ** represents *p* < 0.01 compared with the CNG; # represents *p* < 0.05 and ## represents *p* < 0.01 compared with the ENG; & represents *p* < 0.05 and && represents *p* < 0.01 compared with the CAG; ΔΔ represents *p* < 0.01 compared with the EAG; @ represents *p* < 0.05 compared with the CSG.

Firstly, the MWM test was performed. The spatial learning ability of the mice was evaluated by assessing escape latency. Figure 1A shows that the latency of mice in each group decreased daily through a 5-day learning period. Compared with the CNG group, the latency of the ENG and CAG groups decreased significantly on the 4th day and that of the CSG group increased significantly on the 5th day. Compared with the ENG group, the latency of the EAG group began to decrease significantly on the 4th day, while that of the ESG group began to increase significantly on the 4th day. Compared with the CAG group, the latency of the EAG group decreased significantly on the 5th day, while that of the CSG group increased significantly on the 3rd day. Compared with the EAG group, the latency of the ESG group increased significantly on the 2nd day. Compared with the CSG group, the latency of the ESG group showed a significant decrease on the 5th day.

The number of platform–site crossovers and effective area crossovers was used to assess the spatial memory abilities of the mice. The test was carried out on the 6th day. Figure 1B shows that the number of platform crossings in the ENG group was significantly increased and that the number of platform crossings in the CSG group was significantly decreased compared with the CNG group. Compared with the ENG group, the number of platform crossings in the EAG group increased significantly and the number of platform crossings in the ESG group decreased significantly. Compared with the CAG group, the number of platform crossings in the EAG group increased significantly and the number of platform crossings in the CSG group decreased significantly. Compared with the EAG group, the number of platform crossings in the ESG group was significantly reduced.

#### 3.1.2. Step-Down Test

**Figure 2:**
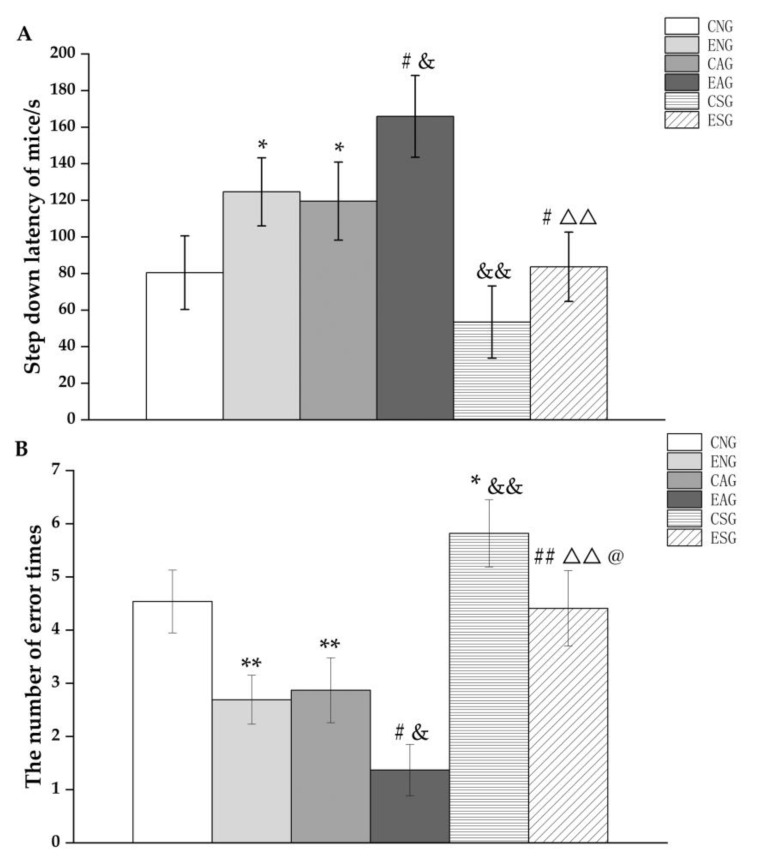
Step-down latency (**A**) and number of errors (**B**) in the step-down test. Note: * represents *p* < 0.05 and ** represents *p* < 0.01 compared with the CNG; # represents *p* < 0.05 and ## represents *p* < 0.01 compared with the ENG; & represents *p* < 0.05 and && represents *p* < 0.01 compared with the CAG; ΔΔ represents *p* < 0.01 compared with the EAG; @ represents *p* < 0.05 compared with the CSG.

Subsequently, the step-down test was performed. From Figure 2A, it can be seen that in terms of step-down latency, the ENG and CAG were significantly increased compared with the CNG group, while the CSG was decreased but did not reach a significant difference. Compared with the ENG, the step-down latency in the EAG was significantly increased, while the ESG was significantly decreased. Compared with the CAG, the step-down latency in the EAG was significantly increased, while the CSG was significantly decreased. Compared with the EAG, the step-down latency in the ESG was significantly decreased. Compared with the CSG group, the step-down latency in ESG was increased but did not reach a significant difference.

It can be seen from Figure 2B that the number of errors was decreased significantly compared with the CNG, the ENG and CAG, while the CSG was significantly increased. Compared with the ENG, the number of errors in the EAG was significantly decreased, while the ESG was significantly increased. Compared with the CAG, the number of errors in the EAG was significantly decreased, while the CSG was significantly increased. Compared with the EAG, the number of errors in the ESG was significantly increased. Compared with the CSG group, the number of errors in the ESG was significantly decreased.

### 3.2. Effects of Aerobic Exercise on the UPR^mt^

#### 3.2.1. Effects of Aerobic Exercise on the mRNA Levels of Key Proteins Regulating the UPR^mt^

**Figure 3:**
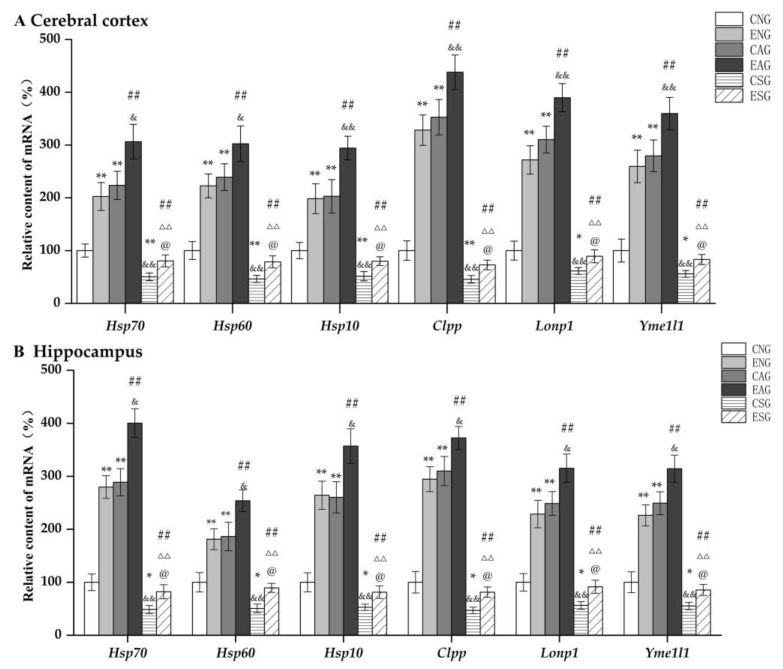
Relative mRNA levels of key proteins regulating the UPR^mt^ in the cerebral cortex and hippocampus. (**A**) Relative content in cerebral cortex. (**B**) Relative content in hippocampus. Note: * represents *p* < 0.05 and ** represents *p* < 0.01 compared with the CNG; ## represents *p* < 0.01 compared with the ENG; & represents *p* < 0.05 and && represents *p* < 0.01 compared with the CAG; ΔΔ represents *p* < 0.01 compared with the EAG; @ represents *p* < 0.05 compared with the CSG.

We first detected the mRNA levels of *Hspa9*, *Hsp60*, *Hsp10*, *Yme1l1*, *Clpp*, and *Lonp1* in the cerebral cortex and hippocampus of mice in each group via QF-PCR. As shown in Figure 3, in the cerebral cortex and hippocampus, compared with the CNG, the mRNA levels of *Hspa9*, *Hsp60*, *Hsp10*, *Yme1l1*, *Clpp*, and *Lonp1* in the ENG and CAG were significantly increased, while those in the CSG were significantly decreased. Compared with the ENG, the mRNA levels of *Hspa9*, *Hsp60*, *Hsp10*, *Yme1l1*, *Clpp*, and *Lonp1* in the EAG were significantly increased, while those in the ESG were significantly decreased. Compared with the CAG, the mRNA levels of *Hspa9*, *Hsp60*, *Hsp10*, *Yme1l1*, *Clpp*, and *Lonp1* in the EAG were significantly increased, while those in the CSG were significantly decreased. Compared with the EAG, the mRNA levels of *Hspa9*, *Hsp60*, *Hsp10*, *Yme1l1*, *Clpp*, and *Lonp1* in the ESG were significantly decreased. Compared with the CSG, the mRNA levels of *Hspa9*, *Hsp60*, *Hsp10*, *Yme1l1*, *Clpp*, and *Lonp1* were significantly increased in the ESG.

#### 3.2.2. Effects of Aerobic Exercise on the Levels of Key Proteins Regulating the UPR^mt^

**Figure 4:**
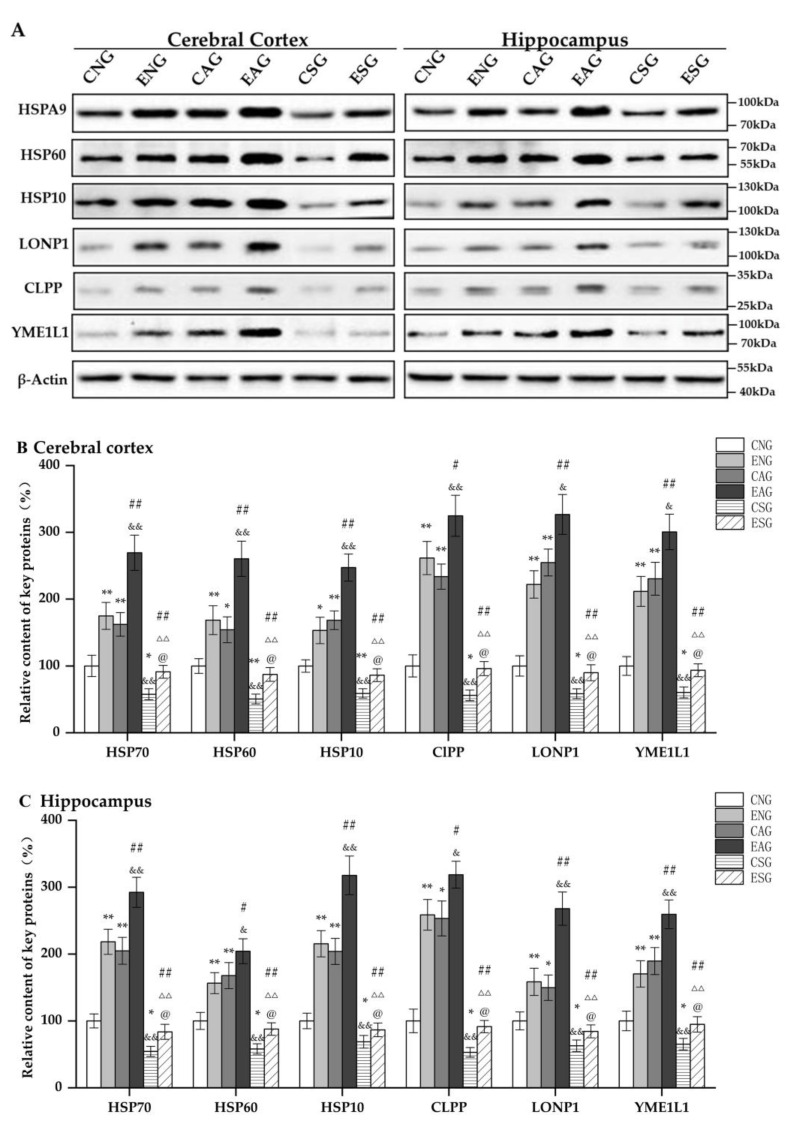
Relative content of key proteins regulating the UPR^mt^ in the cerebral cortex and hippocampus. (**A**) Western blots of key proteins regulating the UPR^mt^ in the cerebral cortex and hippocampus. (**B**) Relative content in cerebral cortex. (**C**) Relative content in hippocampus. Note: * represents *p* < 0.05 and ** represents *p* < 0.01 compared with the CNG; # represents *p* < 0.05 and ## represents *p* < 0.01 compared with the ENG; & represents *p* < 0.05 and && represents *p* < 0.01 compared with the CAG; ΔΔ represents *p* < 0.01 compared with the EAG; @ represents *p* < 0.05 compared with the CSG.

Subsequently, we measured the protein levels of HSPA9, HSP60, HSP10, YME1L1, CLPP, and LONP1 in the cerebral cortexes and hippocampi of mice in each group by Western blotting. As shown in Figure 4, in the cerebral cortex and hippocampus, compared with the CNG, the protein levels of HSPA9, HSP60, HSP10, YME1L1, CLPP, and LONP1 in the ENG and CAG were significantly increased, while those in the CSG were significantly decreased. Compared with the ENG, the protein levels of HSPA9, HSP60, HSP10, YME1L1, CLPP, and LONP1 in the EAG group were significantly increased, while those in the ESG were significantly decreased. Compared with the CAG, the protein levels of HSPA9, HSP60, HSP10, YME1L1, CLPP, and LONP1 in the EAG were significantly increased, while those in the CSG were significantly decreased. Compared with the EAG, the protein levels of HSPA9, HSP60, HSP10, YME1L1, CLPP, and LONP1 in the ESG were significantly decreased. Compared with the CSG, the protein levels of HSPA9, HSP60, HSP10, YME1L1, CLPP, and LONP1 in the ESG were significantly increased.

### 3.3. Effects of Aerobic Exercise on Mitochondrial Autophagy

#### 3.3.1. Effects of Aerobic Exercise on the mRNA Levels of Key Proteins Regulating Mitochondrial Autophagy

**Figure 5:**
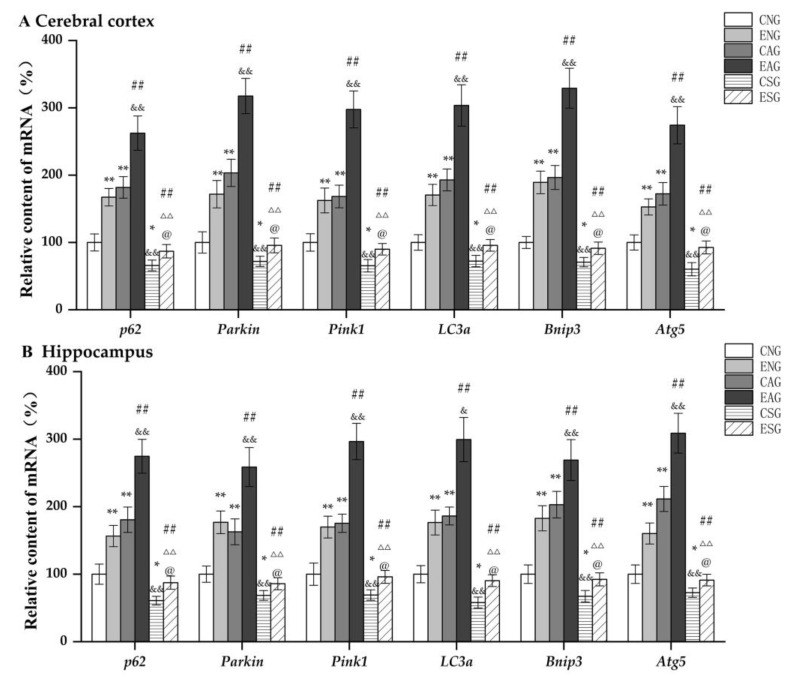
Relative mRNA levels of key mitochondrial autophagy regulators in the cerebral cortex and hippocampus. (**A**) Relative content in cerebral cortex. (**B**) Relative content in hippocampus. Note: * represents *p* < 0.05 and ** represents *p* < 0.01 compared with the CNG; ## represents *p* < 0.01 compared with the ENG; & represents *p* < 0.05 and && represents *p* < 0.01 compared with the CAG; ΔΔ represents *p* < 0.01 compared with the EAG; @ represents *p* < 0.05 compared with the CSG.

We first measured the mRNA levels of *Bnip3*, *p62*, *Atg5*, *Parkin*, *Pink1*, and *LC3a* in the cerebral cortexes and hippocampi of mice in each group by QF-PCR. As shown in Figure 5, in the cerebral cortex and hippocampus, compared with the CNG, the mRNA levels of *Bnip3*, *p62*, *Atg5*, *Parkin*, *Pink1*, and *LC3a* in the ENG and CAG were significantly increased, while those in the CSG were significantly decreased. Compared with the ENG, the mRNA levels of *Bnip3*, *p62*, *Atg5*, *Parkin*, *Pink1*, and *LC3a* in the EAG were significantly increased, while those in the ESG were significantly decreased. Compared with CAG, the mRNA levels of *Bnip3*, *p62*, *Atg5*, *Parkin*, *Pink1*, and *LC3a* in EAG were significantly increased, while those in the CSG were significantly decreased. Compared with the EAG, the mRNA levels of *Bnip3*, *p62*, *Atg5*, *Parkin*, *Pink1*, and *LC3a* were significantly decreased in the ESG. Compared with the CSG, the mRNA levels of *Bnip3*, *p62*, *Atg5*, *Parkin*, *Pink1*, and *LC3a* were significantly increased in the ESG.

#### 3.3.2. Effects of Aerobic Exercise on the Levels of Key Proteins Regulating Mitochondrial Autophagy

**Figure 6:**
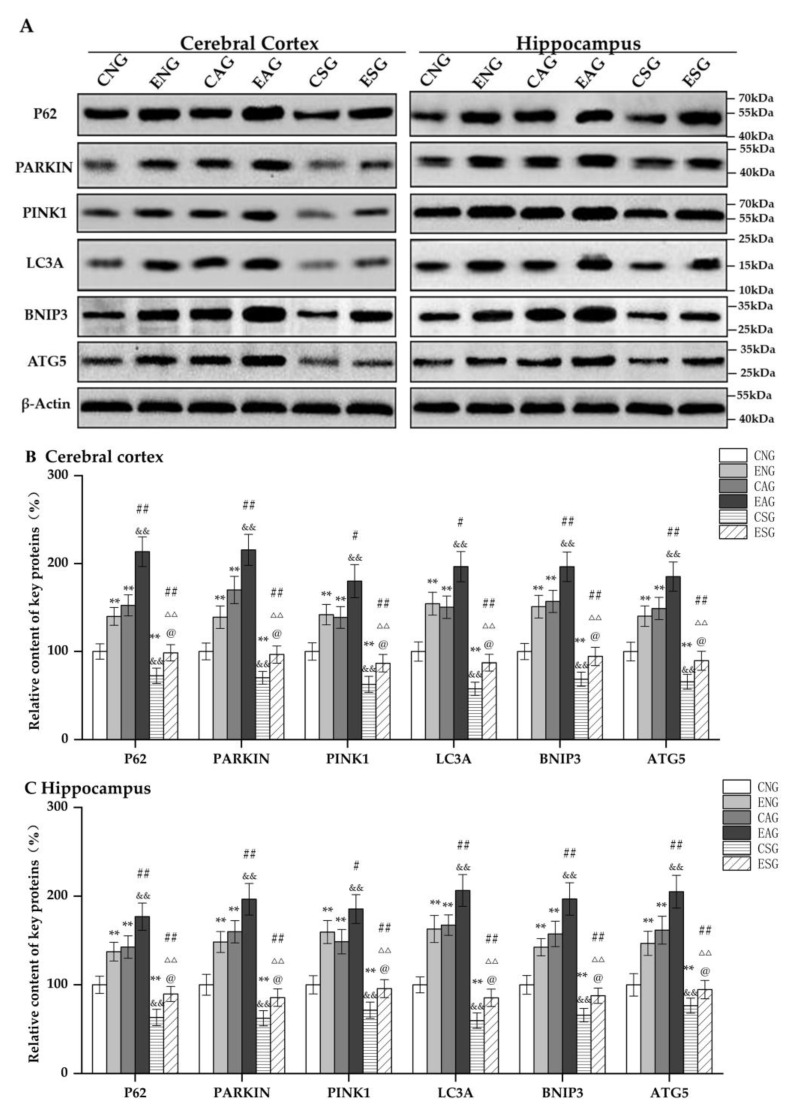
Relative protein content of key mitochondrial autophagy regulators in the cerebral cortex and hippocampus. (**A**) Western blots of key proteins regulating mitochondrial autophagy in the cerebral cortex and hippocampus. (**B**) Relative content in cerebral cortex. (**C**) Relative content in hippocampus. Note: ** represents *p* < 0.01 compared with the CNG; # represents *p* < 0.05 and ## represents *p* < 0.01 compared with the ENG; && represents *p* < 0.01 compared with the CAG; ΔΔ represents *p* < 0.01 compared with the EAG; @ represents *p* < 0.05 compared with the CSG.

Subsequently, we measured the protein levels of BNIP3, P62, ATG5, PARKIN, PINK1, and LC3A in the cerebral cortexes and hippocampi of mice in each group using Western blotting. As shown in Figure 6, in the cerebral cortex and hippocampus, compared with the CNG, the protein levels of BNIP3, P62, ATG5, PARKIN, PINK1, and LC3A in the ENG and CAG were significantly increased, while those in the CSG were significantly decreased. Compared with the ENG, the protein levels of BNIP3, P62, ATG5, PARKIN, PINK1, and LC3A in the EAG were significantly increased, while those in the ESG were significantly decreased. Compared with the CAG, the protein levels of BNIP3, P62, ATG5, PARKIN, PINK1, and LC3A in the EAG were significantly increased, while those in the CSG were significantly decreased. Compared with the EAG, the protein levels of BNIP3, P62, ATG5, PARKIN, PINK1, and LC3A in the ESG were significantly decreased. Compared with the CSG, the protein levels of BNIP3, P62, ATG5, PARKIN, PINK1, and LC3A in the ESG were significantly increased.

### 3.4. Effects of Aerobic Exercise on Mitochondrial Protein Import

#### 3.4.1. Effects of Aerobic Exercise on the mRNA Levels of Key Proteins Regulating Mitochondrial Protein Import

**Figure 7:**
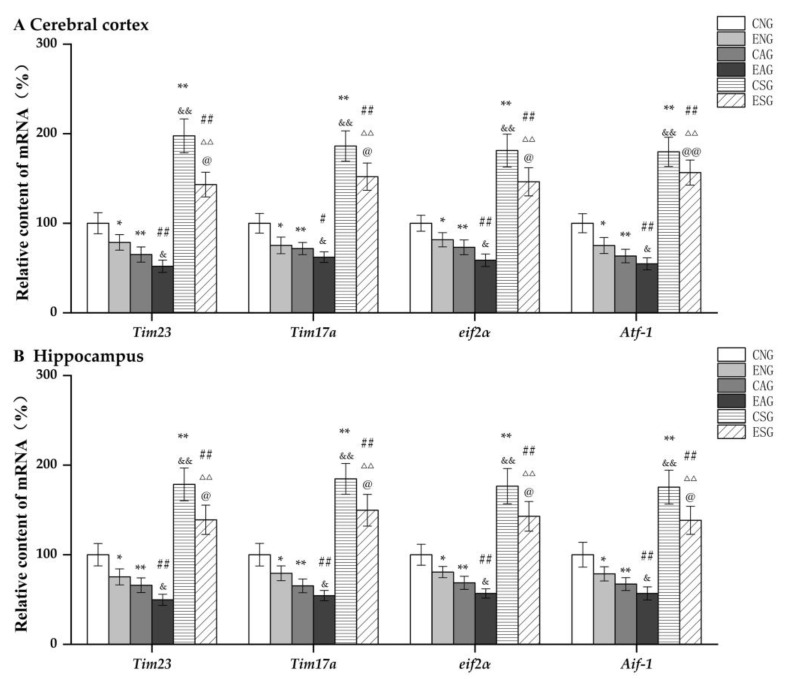
Relative mRNA content of key proteins regulating mitochondrial protein import in the cerebral cortex and hippocampus. (**A**) Relative content in cerebral cortex. (**B**) Relative content in hippocampus. Note: * represents *p* < 0.05 and ** represents *p* < 0.01 compared with the CNG; # represents *p* < 0.05 and ## represents *p* < 0.01 compared with the ENG; & represents *p* < 0.05 and && represents *p* < 0.01 compared with the CAG; ΔΔ represents *p* < 0.01 compared with the EAG; @ represents *p* < 0.05 compared with the CSG and @@ represents *p* < 0.01 compared with the CSG.

We first measured the mRNA levels of *Tim23*, *Tim17A*, *eif2α*, and *Atf-1* in the cerebral cortexes and hippocampi of mice in each group using QF-PCR. As shown in Figure 7, in the cerebral cortex and hippocampus, compared with the CNG, the mRNA levels of *Tim23*, *Tim17A*, *eif2α*, and *Atf-1* in the ENG and CAG were significantly decreased, while those in the CSG were significantly increased. Compared with the ENG, the mRNA levels of *Tim23*, *Tim17A*, *eif2α*, and *Atf-1* in the EAG were significantly decreased, while those in the ESG were significantly increased. Compared with the CAG, the mRNA levels of *Tim23*, *Tim17A*, *eif2α*, and *Atf-1* were significantly decreased in the EAG, while they were significantly increased in the CSG. Compared with the EAG, the mRNA levels of *Tim23*, *Tim17A*, *eif2α*, and *Atf-1* were significantly increased in the ESG. Compared with the CSG, the mRNA levels of *Tim23*, *Tim17A*, *eif2α*, and *Atf-1* were significantly decreased in the ESG.

#### 3.4.2. Effects of Aerobic Exercise on the Level of Key Proteins Regulating Mitochondrial Protein Import

**Figure 8:**
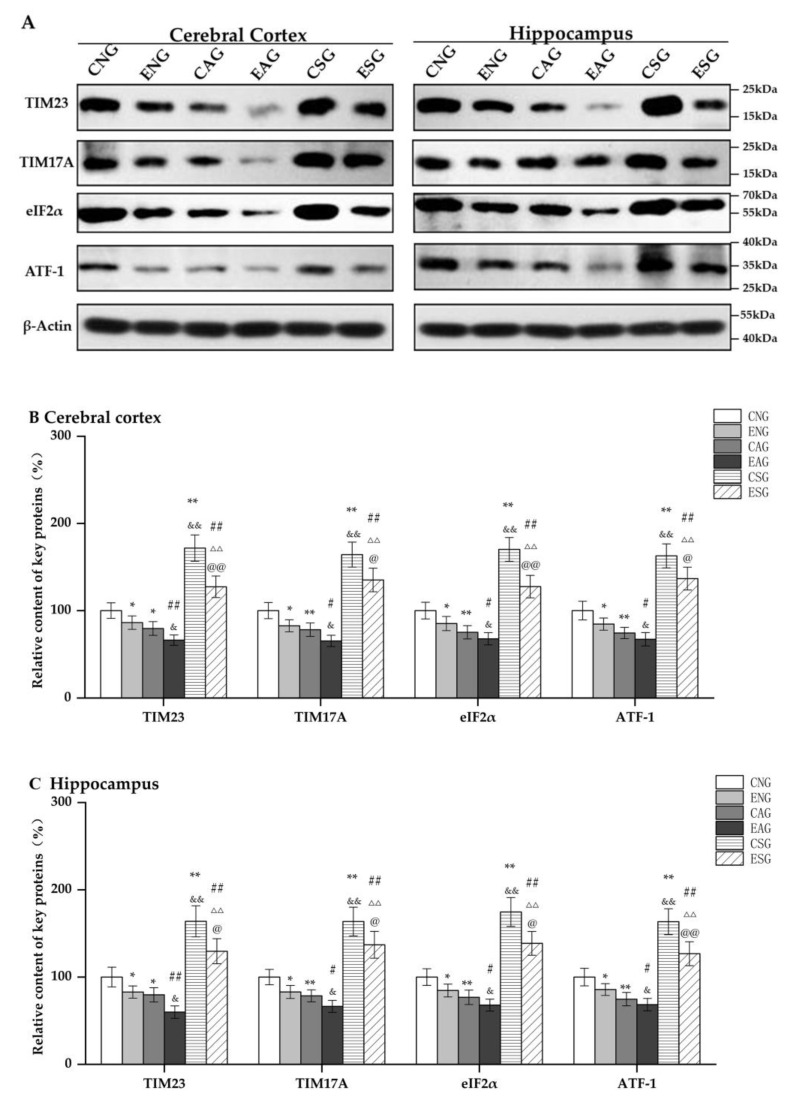
Relative content of key proteins regulating mitochondrial protein import in the cerebral cortex and hippocampus. (**A**) Western blots of key proteins regulating mitochondrial protein import in the cerebral cortex and hippocampus. (**B**) Relative content in cerebral cortex. (**C**) Relative content in hippocampus. Note: * represents *p* < 0.05 and ** represents *p* < 0.01 compared with the CNG; # represents *p* < 0.05 and ## represents *p* < 0.01 compared with the ENG; & represents *p* < 0.05 and && represents *p* < 0.01 compared with the CAG; ΔΔ represents *p* < 0.01 compared with the EAG; @ represents *p* < 0.05 compared with the CSG and @@ represents *p* < 0.01 compared with the CSG.

Subsequently, we measured the protein levels of TIM23, TIM17A, EIF2α, and ATF-1 in the cerebral cortexes and hippocampi of mice in each group through Western blot. As shown in Figure 8, in the cerebral cortex and hippocampus, compared with the CNG, the protein levels of TIM23, TIM17A, EIF2α, and ATF-1 in the ENG and CAG were significantly decreased, while those in the CSG were significantly increased. Compared with the ENG, the protein levels of TIM23, TIM17A, EIF2α, and ATF-1 in the EAG were significantly decreased, while those in the ESG were significantly increased. Compared with the CAG, the protein levels of TIM23, TIM17A, EIF2α, and ATF-1 were significantly decreased in the EAG, while they were significantly increased in the CSG. Compared with the EAG, the protein levels of TIM23, TIM17A, EIF2α, and ATF-1 in the ESG were significantly increased. Compared with the CSG, the protein levels of TIM23, TIM17A, EIF2α, and ATF-1 in the ESG were significantly decreased.

## 4. Discussion

### 4.1. Aerobic Exercise Regulates Mitochondrial Proteostasis and Improves Cognitive Function in AD

Aerobic exercise can improve cognitive function in AD patients by regulating mitochondrial proteostasis [13,14]. The dysfunction and homeostasis imbalance of mitochondria, as the energy centers of the cells, can lead to cell damage and cell death or even neuronal death and cognitive impairment [15]. Research has shown that aerobic exercise is an effective means of regulating mitochondrial function, being able to stimulate the metabolism of mitochondria within cells, regulate their synthesis and degradation, and maintain their proteostasis [16]. Valenzuela et al. summarized in a review [17] that aerobic exercise promotes neurogenesis by increasing exercise-induced metabolic factors (such as ketone bodies and lactic acid) and muscle-derived muscle factors (cathepsin B, iris), thus stimulating the production of neurotrophin (such as BDNF). Simultaneously, aerobic exercise can exert anti-inflammatory effects and improve the redox state of the brain, thereby improving the pathological and physiological characteristics of AD such as amyloid protein β deposition and cognitive impairment [17].

In the MWM test, this study found that the escape latency of mice in both the activation and exercise groups was significantly reduced and the number of platform crossings was significantly increased, while the escape latency of mice in the inhibition group was significantly increased and the number of platform crossings was significantly reduced. In addition, the mice in the exercise-combined activation group had the lowest escape latency level and the highest number of platform crossings, with significant changes. In the step-down test, the activation group and the exercise group mice showed a significant increase in step-down latency, a significant decrease in the number of errors. Conversely, a significant decrease in step-down latency and a significant increase in the number of errors was seen in the inhibition group. In addition, the mice in the exercise-combined activation group had the highest average level of step-down latency and the highest average number of errors, with significant changes. It can thus be suggested that aerobic exercise can improve learning and spatial memory abilities by regulating mitochondrial proteostasis, thereby improving cognitive level.

### 4.2. Aerobic Exercise Improves AD by Activating UPRmt

Mitochondria have their own companion proteins and proteases to enable them to cope with internal misfolded and unfolded proteins [18]. When various mitochondrial stresses are triggered, proteomic dysfunction is perceived and transmitted to the nucleus, activating transcription programs to repair damage; this activated response is called UPR^mt^ [18,19]. UPR^mt^ begins to activate a complex mitochondrial protein quality control (PQC) network. This works by detecting and controlling mitochondrial proteostasis, helping to restore misfolded or unfolded proteins to normal, and ensuring the quality and function of mitochondrial proteomes [19,20]. Of these, two highly conserved chaperone systems are predominantly involved in the folding of mitochondrial precursor proteins, namely, heat shock protein 60 (HSP60) and heat shock protein 70 (HSP70). The chaperone system folds the protein precursor when it is imported into the mitochondria in an unfolded state; this forms a functional structure and exerts its function [21]. In addition, when the mitochondrial protein precursor needs to be hydrolyzed in order to be removed or dissolved and folded again correctly, proteases such as LON peptidase 1 (LONP1) and mitochondrial casein hydrolase P (CLPP, caseinolytic protease) are required [21,22].

Regular aerobic exercise is widely regarded as an effective means of inducing mitochondrial remodeling. This can stimulate mitochondrial biogenesis, activate UPR^mt^, and promote the dynamic balance of the internal environment of the organism [23,24]. At the same time, UPR^mt^ is limited in the mid-to-late stages of AD, and activating UPR^mt^ can reduce Aβ protein deposition in the AD mice and other factors that delay the progression of AD [25]. Xia et al. found that aerobic treadmill training at an intensity of 45 min/day and 5 days/week for 3 months in APP/PS1 mice can regulate UPR^mt^, thereby reducing Aβ deposited in the brain; simultaneously, the authors observed a significant decrease in the expression of β-Site APP cleaving enzyme 1(BACE 1) [26]. In addition, Kang et al. found that aerobic exercise can also regulate the overactivated UPR^mt^ in AD patients, thereby inhibiting inflammatory responses [27].

In this study, the mRNA and protein levels of key proteins such as HSP70 and HSP60, which are regulated by UPR^mt^ levels in the cerebral cortex and hippocampus of mice, were detected by fluorescence quantitative PCR and Western blot experiments. In addition, the mice in the exercise-combined activation group had the highest levels of UPR^mt^-related proteins and showed significant changes. This suggests that aerobic exercise can regulate UPR^mt^ levels in order to maintain mitochondrial proteostasis, thereby improving and delaying AD.

### 4.3. Aerobic Exercise Improves AD by Promoting Mitochondrial Autophagy Levels

Mitochondrial autophagy is a special cellular autophagy process that functions by clearing aging, damaged, and dysfunctional mitochondria; maintaining mitochondrial proteostasis; and promoting cell metabolism and survival. Additionally, it is a key process in mitochondrial self-quality control [28]. The most important regulation of mitochondrial autophagy is the PINK1/PARKIN pathway. This means that in normal mitochondria, PTEN-induced putative kinase 1 (PINK1) will transport complexes into the inner and outer membranes of mitochondria. When mitochondria are damaged, PINK1 will aggregate on the outer membrane surface and recruit Park RBR E3 ubiquitin protein ligase (PARKIN) to initiate mitochondrial autophagy through two pathways [29,30]: (1) PARKIN causes substrate degradation through its counterclaim ligase activity, isolating damaged mitochondria and promoting their phagocytosis by autophagosomes [29,30]. (2) PARKIN-mediated mitochondrial outer membrane hyperubiquitination is recognized by ubiquitin binding junctions, such as Sequestosome 1 (SQSTM1/P62), which recruit damaged mitochondria for autophagy through their interaction with MAP1LC3A (LC3A) [29,30].

Mitochondrial autophagy dysfunction may be a significant feature of AD, and numerous studies have shown that aerobic exercise has a protective effect on mitochondrial dysfunction in AD. After 12 weeks of treadmill exercise for 6-month-old APP/PS1 mice, it was found that aerobic exercise was able to upregulate the ability of mitochondria to recruit PARKIN, enhance mitochondrial autophagy, and accelerate Aβ clearing; moreover, maintaining mitochondrial proteostasis can delay the decline in learning, cognition, and memory abilities in APP/PS1 mice [31]. Zhao et al. found that 12 weeks of aerobic treadmill exercise improved the learning and memory abilities of APP/PS1 mice, reducing Aβ. The deposition of mitochondria also increased mitochondrial autophagy activity, manifesting in a significant decrease in P62 and PINK1 levels, as well as an increase in LC3II and PARKIN levels [32].

In this study, the mRNA and protein levels of key proteins, such as PARKIN, PINK1, and LC3A, which are regulated by mitochondrial autophagy levels in the cerebral cortexes and hippocampi of mice, were detected by fluorescence quantitative PCR and Western blot experiments. It was found that the mitochondrial autophagy level of mice in the activation group and the exercise group significantly increased, while the mitochondrial autophagy level of mice in the inhibition group decreased. In addition, the mice in the exercise-combined activation group had the highest levels of mitochondrial autophagy-related proteins and showed significant changes. This suggests that aerobic exercise is able to improve and delay AD by regulating mitochondrial autophagy levels, thereby maintaining mitochondrial proteostasis.

### 4.4. Aerobic Exercise Improves AD by Reducing Mitochondrial Protein Import

The powerful functions of mitochondria depend on the mitochondrial proteome. Due to the limited ability of mitochondria to synthesize proteins, most mitochondrial proteins are encoded by nuclear genes and synthesized in the cytoplasmic ribosome and transported into mitochondria [29]. The key protein complexes that regulate mitochondrial protein import mainly include the translocator of the outer mitochondrial membrane (TOM) complexes composed of the translocator of the outer mitochondrial membrane 40 (TOM40), the translocator of the outer mitochondrial membrane 20 (TOM20) [29,30,33], the translocator of the inner mitochondrial membrane (TIM) complexes composed of the transformer of the inner mitochondrial membrane complex 17A (TIM17A) and the translator of the inner mitochondrial member 23 (TIM23).

Jung et al. found that treadmill exercise increased the expression of mitochondrial import proteins TOM40, TOM20, and TIM23, thereby improving exercise deficits and mitochondrial protein import disorders [9]. Rui et al. found that aerobic exercise regulates the expression of TOM20 and TIM23 proteins, suggesting that aerobic exercise may be an effective means of regulating mitochondrial import proteins [10]. Zhang et al. found that long-term aerobic endurance training can improve the mitochondrial transport proteins TOM20 and TIM23 and regulate the decrease in the protein expression of mitochondrial chaperones HSP60, HSP70, and HSP10 caused by aging [24].

In this study, the mRNA and protein levels of the key proteins TIM23 and TIM17A regulated by the mitochondrial pathological protein import level in the cerebral cortex and hippocampus of mice were detected through fluorescence quantitative PCR and Western blot experiments. It was found that the mitochondrial pathological protein import level of mice in the activation group and the exercise group significantly decreased, while the mitochondrial pathological protein import level of mice in the inhibition group decreased. In addition, the mice in the exercise-combined activation group had the lowest levels of mitochondrial pathological protein import regulation key proteins, and there were significant changes. Improvement in mitochondrial pathological protein import levels through activation and aerobic exercise is thus suggested. In the research on the regulation of mitochondrial channel protein levels, such as TIM23, via exercise, there have been controversies due to specific training plans, the course of disease in AD mice, and individual differences in AD mice. Nevertheless, it is suggested that aerobic exercise can maintain mitochondrial proteostasis and improve and delay AD by regulating mitochondrial protein import levels.

## 5. Conclusions

In summary, aerobic exercise can improve cognitive function levels and delay AD symptoms in APP/PS1 mice by regulating mitochondrial proteostasis. The specific mechanisms are as follows: (1) aerobic exercise reduces the accumulation of unfolded or misfolded mitochondrial proteins by activating UPR^mt^ and maintaining mitochondrial proteostasis; (2) aerobic exercise promotes mitochondrial autophagy, removes damaged or abnormal mitochondria, and maintains mitochondrial proteostasis; and (3) aerobic exercise reduces the excessive import of mitochondrial proteins, reduces the burden of mitochondrial chaperones and proteases, and maintains mitochondrial proteostasis.

## Figures and Tables

**Table 1:** Twelve-week treadmill training program.

Week	Running Speed (m/min)	Training Time (min)
1–3	12	30
4–6	13	40
7–9	14	50
10–12	15	60

**Table 2:** Primer sequences for Q-PCR analysis of tissue samples.

Gene	Upstream Primer	Downstream Primer
*Hspa9*	5′-CAAGTCAGATTGGAGCAT-3′	5′-CATTGAAATAAGCAGGGA-3′
*Hsp60*	5′-GCACTGGCTCCTCATCTC-3′	5′-CACAGTTCTTCCCTTTGG-3′
*Hsp10*	5′-AGTTTCTTCCGCTCTTTG-3′	5′-ACTCTTTCCTTTCCCTCC-3′
*Yme1l1*	5′-TTAAGGGACCTTGGATTA-3′	5′-AAGGACTGTGCCGAAATA-3′
*Clpp*	5′-TCTGTTGTCTCGCCTTGC-3′	5′-CCGTCTGCTCCACCACTA-3′
*Lonp1*	5′-TCCTCACCTGCCGCTCAT-3′	5′-GAAGACGCCAACATAGGG-3′
*Bnip3*	5′-GCTGAAATAGACACCCAC-3′	5′-GACTTGACCAATCCCATA-3′
*p62*	5′-ATGAGTGGATTTAACTTTG-3′	5′-CACTTGAGATGGCATTGGT-3′
*Parkin*	5′-GTGGTTGCTAAGCGACAG-3′	5′-GTTGTTCCAGGTCACAGTTT-3′
*Pink1*	5′-GGCTGATCGAGGAGAAGCA-3′	5′-CATCGAGTGTCCAGTGGGT-3′
*LC3a*	5′-CAGCATGGTGAGCGTCTC-3′	5′-CCGAAGGTTTCTTGGGAG-3′
*Tim23*	5′-ATTGAAGGAAACCCAGAG-3′	5′-CTAGAGTATTAGCCCAAAGTG-3′
*Tim17A*	5′-CCGAGGAAGTTTGACAGC-3′	5′-CACCGCTAGTGATGGAGTT-3′
*eIF2α*	5′-CGCCATGTTGCTGAGGTA-3′	5′-GCATCGTAGGCACCGTAT-3′
*ATF-1*	5′-ACGGAGCCTTACAGTTGG-3′	5′-TCGGGACGAGTATCTGCT-3′
*β-Actin*	5′-GTTGACATCCGTAAAGAC-3′	5′-TAGGAGCCAGGGCAGTAA-3′

## Data Availability

The data presented in this study are available on request from the corresponding author.
